# Management of a Long-Standing Huge Goiter During a Humanitarian Mission: A Case Report

**DOI:** 10.7759/cureus.39365

**Published:** 2023-05-23

**Authors:** Cheikh Ahmédou Lame, Mehmet Atila, Bertin Dembele, Michael K Obeng

**Affiliations:** 1 ENT Department, Hopital Principal de Dakar, Dakar, SEN; 2 Center for Aesthetic Plastic Surgery, Medical Inn, Düsseldorf, DEU; 3 Plastic Surgery and Oncology Department, Hôpital de Dermatologie de Bamako, Bamako, MLI; 4 Plastic Surgery Department, University of Pittsburgh School of Medicine, Los Angeles, USA

**Keywords:** africa, humanitarian mission, thyroidectomy, endemic goiter, giant goiter

## Abstract

Giant goiters are still encountered in low-income countries where diagnosis and management remain challenging. The authors report a case of an endemic giant goiter, treated during a humanitarian mission. A 50-year-old female with no particular history presented during a humanitarian mission with a giant nontoxic goiter evolving for 30 years. She underwent a total thyroidectomy removing a 3 kg thyroid gland. The postoperative period was uneventful. Giant goiters are not exceptional in goiter-endemic areas. Diagnostic and surgical management do not require particular technology. Surgery remains feasible even in countries with limited resources.

## Introduction

Thyroid diseases are one of the most common endocrine disorders worldwide. Endemic goiter due to iodine deficiency remains a public health concern in sub-Saharan Africa. In these areas, given the lack of available health care and socioeconomic issues, many patients often neglect their diseases and can present with enormous goiters. These massive goiters may result in significant pressure symptoms like dyspnea and dysphagia and sometimes be the cause of stigma and rejection. Surgical removal is the treatment of choice in such cases. It provides instant relief from the effects of pressure and ensures good cosmetic results.

However, in developing countries, due to delayed diagnosis and lack of qualified human resources, surgical management of these goiters remains challenging [1-3]. Here, the authors report a case of endemic giant goiter operated during a humanitarian mission.

## Case presentation

During a surgical humanitarian mission in a low-income Sub-Saharan African country, a 50-year-old female, with no past medical history, who was not taking any medications, presented with a large cervical mass evolving for nearly 30 years. The patient complained of heaviness-like pain without dysphonia or dysphagia. She reported feeling moderate dyspnea when walking or during household activities. Aesthetic and social embarrassment was very important and associated with ostracism.

**Figure 1:**
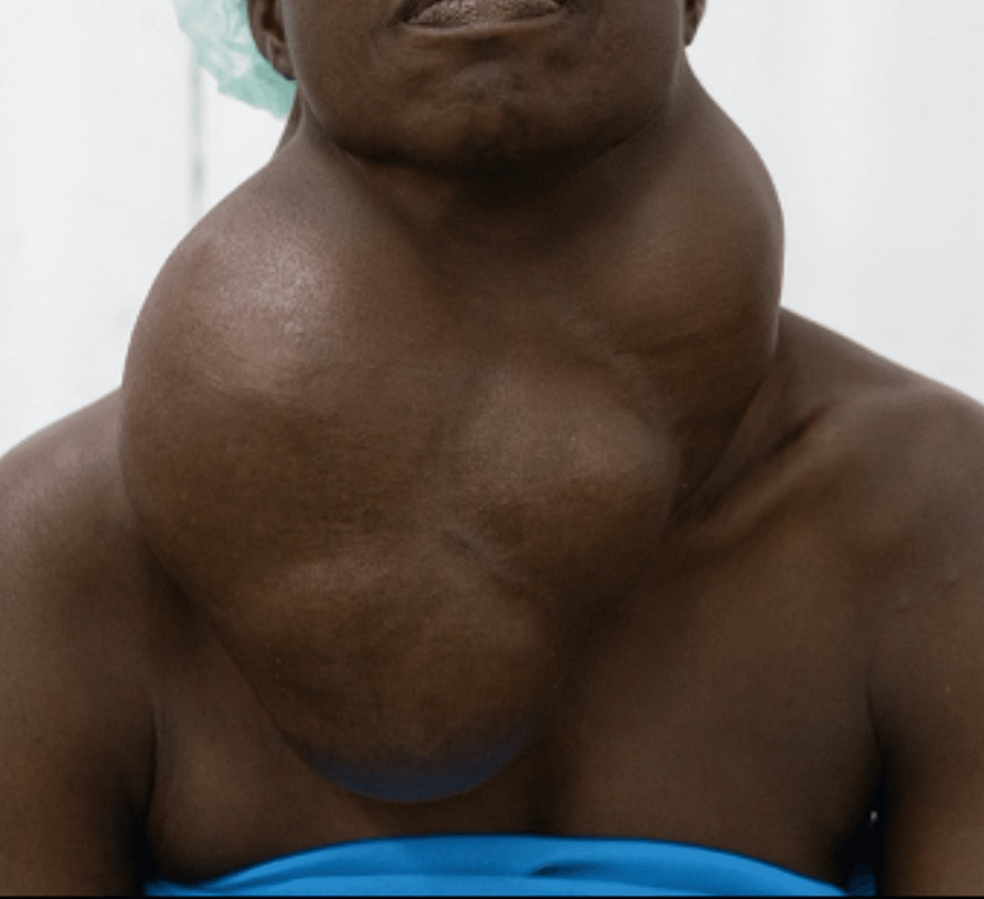
Anterior view of patient with huge multinodular goiter

On physical examination, she had a huge hypertrophy of the thyroid gland, occupying the whole neck, and overflowing laterally (Figure 1) measuring 23 cm x 17 cm x 15 cm. This firm goiter was painless, not fixed to the underlying structures, and without thrill or vascular murmur. There was no proptosis, no tachycardia, and no palpable lymph nodes. From the clinical findings, the diagnosis of huge, nontoxic, multinodular goiter was retained. 

**Figure 2:**
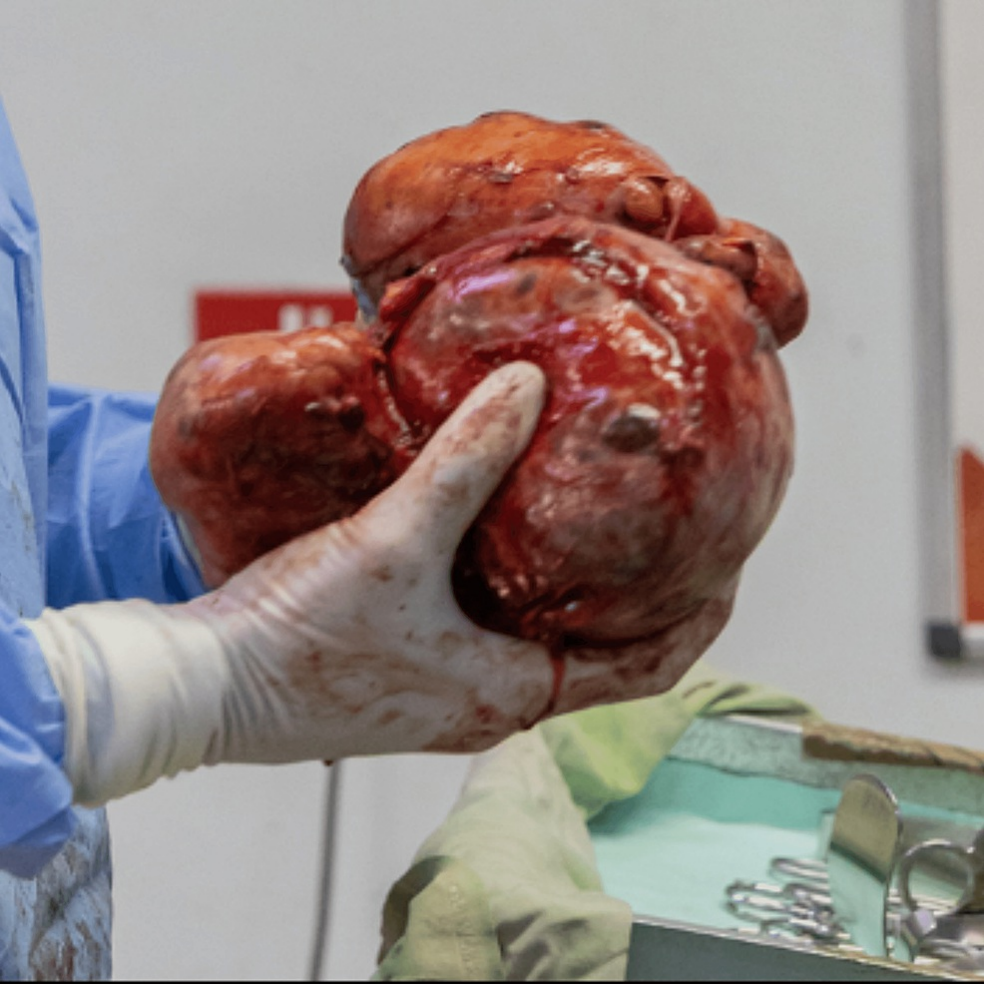
Total thyroidectomy specimen weighing 3 kg

Total thyroidectomy was performed under general anesthesia after the identification and preservation of recurrent laryngeal nerves and parathyroid glands. It allowed the excision of a bulky, firm, multi-nodular thyroid weighing 3 kg (Figure 2).

Postoperative recovery was uneventful without dyspnea or muscle cramps. The patient was discharged three days later, with levothyroxine replacement therapy (100 micrograms per day), and placed under the supervision of the general practitioner.

## Discussion

Giant goiters can still be encountered in goiter-endemic areas [1-3]. These enormous goiters exceeding 500 grams are extremely rare and often found in sub-Saharan Africa and in some Asian countries [1-3]. Their occurrence in developing countries increases the complexity of the management [4].

In these places, the management of giant goiters often comes up against a diagnostic delay, a lack of qualified human resources, and an insufficient technical platform [2,3,5,6]. This was the case of our patient who lives in a remote area of a sub-Saharan African country marked by difficult access to healthcare facilities. 

Technology to support medical decision-making is often lacking in these places [2]. For our patient, in the context of a humanitarian mission, and in the absence of biology and imaging assessments, we contented ourselves with anamnestic data and physical examination to make a diagnosis of huge non-toxic multinodular goiter and decide for intervention.

Total thyroidectomy is the best treatment modality for giant goiter [7]. It does not require any special conditions other than a sterile operating room, lights, and the availability of conventional soft tissue surgery instruments [2,5]. Anesthesia for patients with huge goiter can face intubation difficulty [8-11]. This exigent surgery is very challenging. It imposes perfect control of the thyroidectomy technique [9]. Morbidity and mortality linked to this surgery remain significant whatever the working conditions [2,12]. In the described case, no postoperative complications developed. The patient was discharged home on the hospital day 3.

## Conclusions

Giant endemic goiters still exist in sub-Saharan Africa. Surgery is the main treatment modality in such cases. Subtotal thyroidectomy, with its long-term risk of recurrence, is an option but total thyroidectomy is the preferred one. The volume and the weight of these huge goiters might involve some surgical difficulties. However, the procedure does not require any particular technology and can be performed regardless of the work context.
